# Top-down fabrication of high-uniformity nanodiamonds by self-assembled block copolymer masks

**DOI:** 10.1038/s41598-019-43304-5

**Published:** 2019-05-06

**Authors:** Jiabao Zheng, Benjamin Lienhard, Gregory Doerk, Mircea Cotlet, Eric Bersin, Harrison Sejoon Kim, Young-Chul Byun, Chang-Yong Nam, Jiyoung Kim, Charles T. Black, Dirk Englund

**Affiliations:** 1 Department of Electrical Engineering and Computer Science, Massachusetts Institute of Technology, Cambridge, Massachusetts 02139 United States; 2 Center for Functional Nanomaterials, Brookhaven National Laboratory, Upton, NY 11973 USA; 3 Department of Materials Science and Engineering, The University of Texas at Dallas, 800 West Campbell Road, Richardson, Texas 75080 USA

**Keywords:** Molecular self-assembly, Nanoparticles, Single photons and quantum effects, Quantum optics

## Abstract

Nanodiamonds hosting colour centres are a promising material platform for various quantum technologies. The fabrication of non-aggregated and uniformly-sized nanodiamonds with systematic integration of single quantum emitters has so far been lacking. Here, we present a top-down fabrication method to produce 30.0 ± 5.4 nm uniformly-sized single-crystal nanodiamonds by block copolymer self-assembled nanomask patterning together with directional and isotropic reactive ion etching. We show detected emission from bright single nitrogen vacancy centres hosted in the fabricated nanodiamonds. The lithographically precise patterning of large areas of diamond by self-assembled masks and their release into uniformly sized nanodiamonds open up new possibilities for quantum information processing and sensing.

## Introduction

Over the past two decades, solid-state defects have emerged as one of the leading systems for a wide variety of quantum technologies. Solid-state hosts such as diamond or silicon carbide are well studied, and a wide spectrum of fluorescing crystal defects have been identified and characterized^1–5^. In particular, the diamond nitrogen vacancy (NV) centre with its optically-addressable long-lived spin system is well suited for applications ranging from quantum networks^6,7^ to quantum sensors^8^. Research interest has been growing considerably in developing other atomic emitters, such as the group-IV vacancy centres, possessing similar characteristics but with improved spectral stability^9–12^.

Nanodiamonds (ND) hosting such colour centres are promising for various biological^13^ and quantum^14–16^ technologies, thanks in part to their compatibility with biologically active tissue and with common surface modification techniques^13,17^. Furthermore, they have been employed for laser trapping techniques^18–20^ and scanning tip microscopy^21–23^. In comparison with bulk diamond, NDs offer nanometre-scale spatial positioning and compatibility with optical levitation. Typical commercialized techniques for ND fabrication include detonation, laser ablation, balling milling, high-pressure high-temperature (HPHT) growth, and chemical vapor deposition (CVD) growth^24^. However, these methods lack control over size or aggregation of the resulting NDs^14^. A reliable fabrication method of non-aggregated, uniform-sized, monocrystalline NDs with incorporated single colour centres is still challenging.

Control over the ND size uniformity is an important figure of merit for applications that involve building nano-hybrids^25^, self-assembly processes^26,27^, or integration with quantum emitters created via ion implantation^28^. Controlling the ND size requires precise tuning of the detonation, milling, or growth process and is usually followed by an extra ultracentrifugation step. Furthermore, the separation of individual NDs^29^ is an important requirement to prepare ND samples for subsequent integration with photonic nanostructures^30^ or biological tissue^31^. Traditional methods require stabilizing agents which can introduce contamination during the sample preparation^32^. Furthermore, control of doping concentration in NDs is necessary for applications such as nanodiamond electronics^33^ and optical levitation of NDs^34^. Methods based on ion implantation^28^ or detonation synthesis^35^ are part of ongoing research.

Solutions to these fabrication challenges have been explored, and while progress has been made, many problems still remain. A fabrication approach for reactive ion etching of NDs using a sputtered gold mask with mean particle diameters of 50 nm has been demonstrated and resulted in NDs hosting implanted NVs with spin coherence times, T_2_, exceeding 200 µs^36^. However, such sputtered gold masks suffer from significant non-uniformity, and the mechanical process utilized to release diamond nanopillars from the parent diamond causes loss of material. Another option to fabricate NDs is to use a CVD grown diamond membrane with a delta-doped NV layer^37^. Electron beam lithography (EBL) and plasma etching can then be used to control the ND size. This technique enabled the demonstration of NVs in NDs with 200 nm diameter on average and a T_2_ exceeding 700 µs. However, the EBL fabrication of the mask is not scalable, and the resultant particles exceed the size requirements for many applications.

Here, we demonstrate large-scale parallel fabrication of non-aggregated, uniform-sized, monocrystalline NDs hosting single NV centres. The fabrication technique starts with CVD grown high-purity single-crystal diamond. It leverages the scalability of block copolymer (BCP) self-assembly combined with sequential infiltration synthesis to define nanometre-sized etching masks across an arbitrarily large diamond sample. A directional plasma etching step defines the dimensions of the NDs. An isotropic plasma etching step releases the NDs. We confirmed single-photon emission from single NVs hosted in the fabricated NDs. Statistics of the diameter of the released NDs indicate a mean diameter of about 30 nm with a variance of 5.4 nm.

## Results

**Figure 1:**
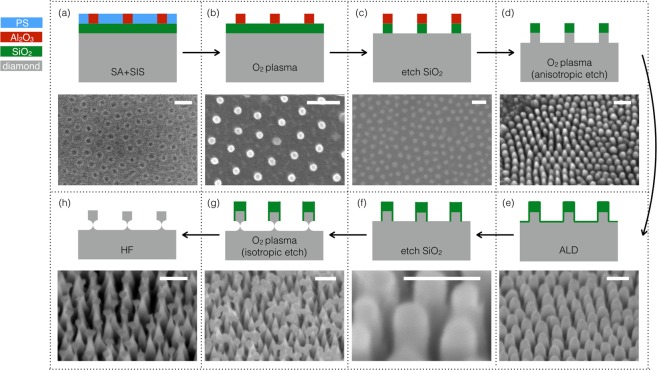
Fabrication process flow in 8 steps, with colour legend at upper left indicating the material of each layer. Each subfigure corresponds to one fabrication step, with schematic side-view diagram, and text showing the specific process carried out in the particular step, together with SEM images taken after that step, showing resultant device morphology. All scale bars indicate 100 nm. Note that images for steps (**a**–**c**) are top view, and images for other steps were obtained with samples tilted at 45 degrees. (**a**) Polished bulk single crystal diamond, coated with ~30 nm SiO_2_ layer using PECVD, with AlO_x_ hexagonal dot array patterned by BCP film using spin coating, SA and SIS. (**b**) Gentle O_2_ plasma treatment removes the polymer content in the BCP film, leaving AlO_x_ hexagonal dot array pattern. (**c**) Directional plasma etching of SiO_2_, transferring the dot array pattern to SiO_2_ layer. (**d**) Directional O_2_ plasma etching of diamond, transferring the dot array pattern to bulk diamond, forming diamond pillars arranged in hexagonal array. (**e**) ALD of ~2 nm SiO_2_ coating to protect the sidewall of etched diamond pillars. (**f**) A short directional plasma etch removes SiO_2_ from bottom surfaces of the open gaps between diamond pillars. (**g**) The sample is exposed to a quasi-isotropic O_2_ plasma to undercut the diamond pillars. (**h**) The bulk diamond is immersed in HF acid to remove the residue SiO_2_ layer.

**Figure 2:**
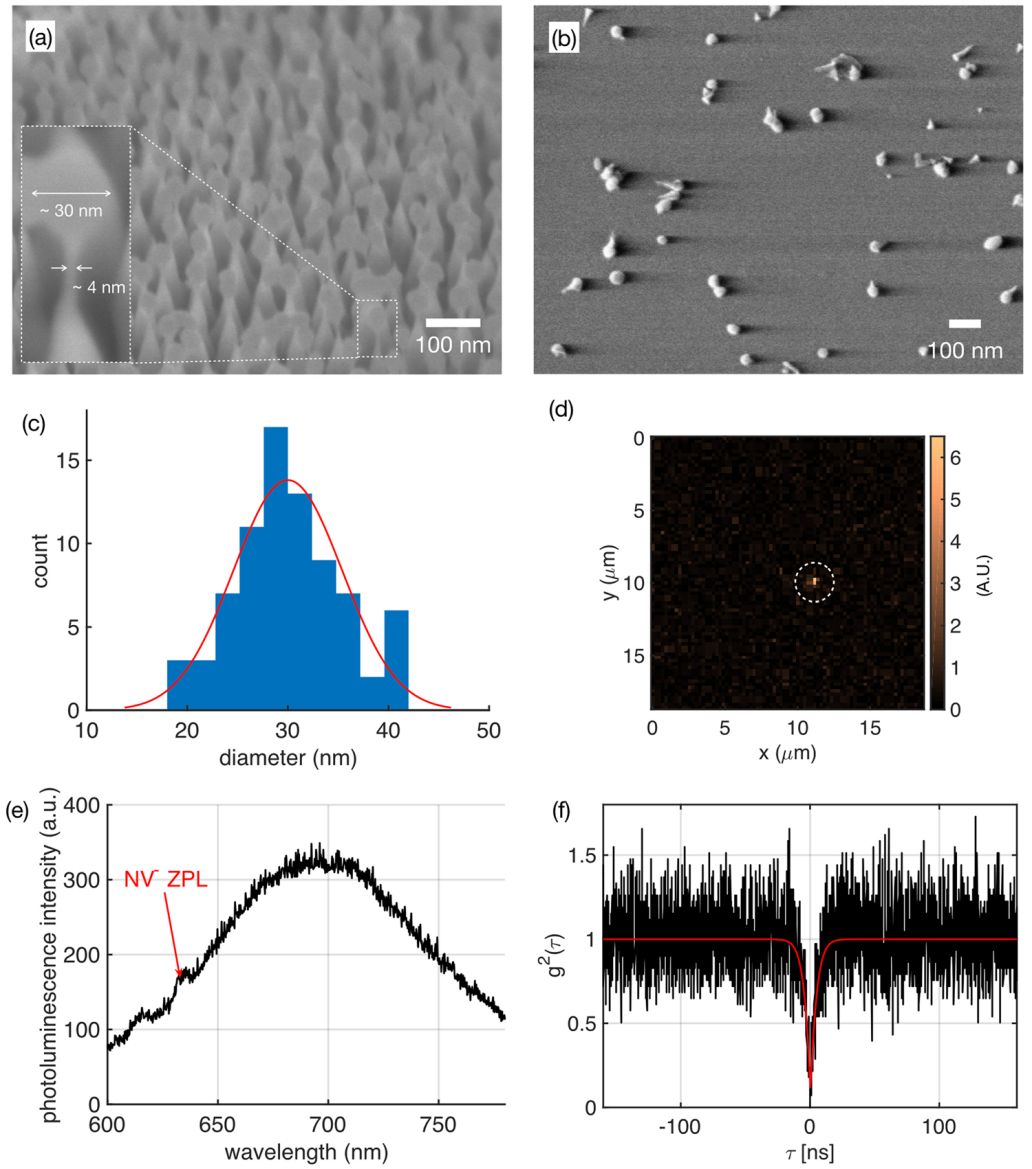
(**a**) SEM of fabricated NDs before release, featuring ~30 nm sized NDs sitting on ~4 nm diamond pedestals. (**b**) SEM of NDs released on glass substrate. (**c**) Particle size distribution histogram of NDs, determined from large scale SEM imaging and subsequent image processing to detect the size of the NDs in the SEM image. The red curve shows a Gaussian fit to the histogram with a mean diameter of 30 nm and a variance of 5.4 nm. (**d**) Optical confocal scanning image of the NDs released on glass substrate. The bright spot (white dashed circle) indicates an ND hosting a single NV colour centre. (**e**) Photoluminescence spectrum collected from the bright spot shown in (**d**), with the characteristic NV zero phonon line (ZPL) at 636.5 nm and a broad phonon sideband. (**f**) Second-order autocorrelation histogram of the collected emission from the bright spot in (**d**), showing g^2^(0) of 0.08 indicating single photon emission (g^2^(0) < 0.5).

Our ND fabrication process starts with commercially available bulk monocrystalline diamond (Element Six) grown by a microwave-assisted CVD process. We use IIA optical grade monocrystalline diamond (size 3 × 3 × 0.5 mm^3^) with a nitrogen concentration of less than 5 ppm (corresponding to ~12 nitrogen atoms for 30 nm diameter diamond spheres). We deposit a ~30 nm SiO_2_ layer by plasma-enhanced chemical vapor deposition (PECVD) as an etch stop layer. As outlined in Fig. 1, the fabrication process proceeds as follows: (a) The mask array in a hexagonal lattice configuration is produced by BCP self assembly (SA), followed by sequential infiltration synthesis (SIS)^38,39^ to selectively load dots with AlO_x_ to produce a hard etch mask that can withstand the subsequent reactive ion processing steps, see Supplemental Information (SI) for details. (b) Oxygen plasma reactive ion etching (RIE) removes the polymer, leaving only the AlO_x_ dots array pattern. (c) Dry etching of SiO_2_ using SF_6_ and oxygen plasma transfers the hexagonal dot array pattern into the SiO_2_ etch stop layer. (d) Directional oxygen plasma etching transfers this SiO_2_ dot array pattern into the diamond, producing a hexagonal array of diamond pillars with a height tunable by the directional oxygen RIE duration, targeted here to 30 nm. (e) Atomic layer deposition (ALD) of SiO_2_ protects the diamond pillar sidewalls. (f) Directional plasma etching of the SiO_2_ using an SF_6_ and O_2_ gas mixture removes the SiO_2_ layer at the non-sidewall surfaces. (g) Quasi-isotropic oxygen plasma etching^40^ partially undercuts the bottom surface of the diamond pillars, see SI for details. At the end of this process, the ~30 nm sized diamond nanocrystals are nearly free-standing on ultrathin (~4 nm) pedestals, as shown in the close-up scanning electron micrograph (SEM) in Fig. 2a. (h) Finally, hydrofluoric (HF) acid removes the SiO_2_ masks and the passivation layers, leaving the diamond nanocrystal for characterization or subsequent releasing steps.

### ND size distribution

To characterize the fabricated NDs, we released the NDs on commercially available glass coverslips. We then performed large area SEM imaging of the NDs, as exemplified in Fig. 2b. Customized image processing detects single NDs on the SEM images and estimates the dimensions based on the image contrast. We evaluated over 100 NDs and visually confirmed the boundary detection results. The resultant ND diameter statistics are fitted to a Gaussian distribution curve, as shown in Fig. 2c in red. The Gaussian fit yields a mean diameter of 30.0 nm with the 95% confidence interval from 28.8 nm to 31.2 nm, and a variance of 5.4 nm with the 95% confidence interval from 4.7 nm to 6.4 nm.

### Optical characterization

The optical measurements were performed in a home-built confocal setup with 532 nm laser excitation and an excitation power of ~3 mW after the objective. The collection path can either be directed through a 690/40 nm notch filter to a single-photon avalanche photodiode (SPAD), or through a 561 nm long pass filter to an optical spectrometer.

Figure 2d shows a confocal scanning map of NDs released on a coverslip. The scan reveals a single bright photoluminescent spot; the corresponding spectrum collected from the bright spot, as shown in Fig. 2e, indicates a room temperature NV emission spectrum in its negative charge state with the characteristic zero phonon line (ZPL) at 636.5 nm.

Second-order autocorrelation measurements were conducted with a home built confocal setup with a 594 nm laser at ~200 µW excitation power on the sample to excite the NV^−^ charge state, and a 635 nm long pass filter followed by a Nikon oil immersion objective with numerical aperture (NA) of 1.3. The second-order autocorrelation measurements (g^(2)^(0)~0.08) in Fig. 2f indicate emission from a single NV centre with an emission of up to 80,000 single photons per second, recorded with SPADs.

## Discussion

We presented a top-down fabrication technique to produce non-aggregated monocrystalline NDs with a uniform size. The presented method enables the fabrication of NDs with a mean diameter of 30 nm and a variance of 5.4 nm. The process is performed on a IIA grade bulk diamond with a known nitrogen dopant concentration of 5 ppm. Second-order autocorrelation measurements confirm the presence of single quantum emitters in the ND.

This method can be applied to bulk diamond, diamond with tailored dopant properties, or isotopically purified diamond to produce NDs with the same dimensional properties^41,42^. Doped NDs are attractive materials for the study of medical oncology^43^, electrochemistry^44,45^, diamond electronics^46^, and superconductivity^47,48^. Diamond enriched with ^12^C can enable increased coherence times of hosted spin systems due to the limited number of interfering spin impurities in the host material^42^.

The produced ND dimensions are determined by the mask patterning technique and the vertical etching step, shown in Fig. 1(d). This scalable mask patterning and etching technique is compatible for producing NDs with a wide range of dimensions and aspect ratios. The size of the dot masks obtained by BCP SA followed by SIS can be tuned from ~10 to ~30 nm in diameter by changing the SIS cycle number^39,49^, nanoimprint lithography can be used^50^ for ~30–1000 nm in size, and traditional optical lithography is applicable for mask sizes beyond 1 µm. The vertical height of the NDs can be independently controlled by the dwell time of the vertical etching in the processing step shown in Fig. 1(d).

Upon completion of the fabrication process, the NDs are non-aggregated, since the nanomask defined in the BCP SA process naturally separates one domain from another. The quasi-isotropic plasma undercut process exposes a large surface area and allows further surface treatments of the NDs. Chemical treatments such as surface modifications to minimize aggregation^27,32^ or surface functionalization for bio-binding^51^ are important prerequisites for a wide range of research processes and topics^52–54^. Such surface treatments are compatible with the underlying ND fabrication method. Furthermore, different surface terminations can change the quantum properties of NV centres^55^, such as a nitrogen plasma treatment^56^, or can stabilize the charge state of NV centres^57^, such as a fluorine-based or oxygen-based plasma treatment.

The exposed surfaces of the resulting NDs provide an opportunity to grow conformal coatings for protection or functionalization before harvesting. For example, ALD of isotopically purified SiO_2_ can be used to “package” the NDs with fewer spin impurities to improve the spin coherence of the hosted NVs. Such an ALD process can be achieved with precursors such as silane^58^, for which isotopically enriched sources are commercially available thanks to the well-developed silicon growth process^59^. The oxidant for the SiO_2_ ALD process can originate from naturally occurring oxygen. Naturally occurring oxygen has an abundance of 99.76% nuclear-spin-free ^16^O, higher than the naturally occurring 98.93% abundance of nuclear-spin-free ^12^C in diamond.

The estimated production yield can be scaled up using a larger area and iterating in the next depth layer of the parent diamond. This process promises large-area fabrication of NDs with size and shape uniformity, thanks to the scalability of the BCP SA process. As demonstrated in this work, the ND production yield scales with the parent diamond area size. The 70 nm pitch used in the BCP SA nanomask template translates to ~2 × 10^8^ NDs with ~30 nm in diameter for every 1 × 1 mm^2^ diamond area. This corresponds to a production yield of ~9.89 ng/mm^2^ measured in weight per unit area of the parent bulk diamond. However, the overall mass of nanodiamonds produced in this planar patterning method is of course much lower than it would be for 3D fabrication methods, such as ball-milling, though these 3D fabrication methods are unable to achieve the same size uniformity^60^.

The potential impact of the presented ND fabrication technique goes beyond size uniformity and non-aggregation. The quasi-isotropic plasma undercut may induce less strain on the NDs compared to other methods for top-down fabrication of nanodiamonds^36,37^, though further studies are required. It would also be relevant to study the spin coherence times of group IV vacancy centres hosted in NDs produced by this process^11^. The spin coherence time T_2_ of the silicon vacancy (SiV) centre is limited by the phonon-induced relaxation between two orbital ground states that are split by ~47 GHz^61^. NDs with diameters below half the corresponding phononic wavelength (~127 nm) may suppress the coupling of these phonon modes to prolong the SiV spin coherence times^62^. At the same time, it is important to minimize stress/strain in the NDs to maintain the defect centre’s inversion symmetry such that their optical properties are preserved^63^.

## Conclusions

We realized a wide-area fabrication process in a top-down approach to produce non-aggregate monocrystalline NDs with high size uniformity. Additional optical characterization confirmed the existence of single NVs hosted in the NDs by probing their photoluminescence spectra and photon statistics. The fabrication technique applies to a broad range of engineered bulk diamond, including for example isotopically purified substrates. ^12^C enriched diamond hosted spin systems have been shown to greatly extend the NV electron spin coherence time^36,37^.

## Supplementary information

Supplementary Information for “Top-down fabrication of high-uniformity nanodiamonds by self-assembled block copolymer masks”

## Data Availability

The data that support the findings of this study are available upon reasonable request.
