# Supplementary material for: Top-down fabrication of high-uniformity nanodiamonds by self-assembled block copolymer masks
Source: Sci Rep. 2019 May 6;9:6914. doi: 10.1038/s41598-019-43304-5 (PMC6502864; doi:10.1038/s41598-019-43304-5)
Supplement: Supplementary file 1 — Supplementary Information for “Top-down fabrication of high-uniformity nanodiamonds by self-assembled block copolymer masks” [file 41598_2019_43304_MOESM1_ESM.pdf]

Supplementary Information for “Top-down fabrication of high-uniformity nanodiamonds by self-assembled block copolymer masks”

Jiabao Zheng[1]*, Benjamin Lienhard[1], Gregory Doerk[2], Mircea Cotlet[2], Eric Bersin[1], Harrison Sejoon Kim[3], Young-Chul Byun[3], Chang-Yong Nam[2], Jiyoung Kim[3], Charles T. Black[2], Dirk Englund[1]*

1 Department of Electrical Engineering and Computer Science, Massachusetts Institute of Technology, Cambridge, Massachusetts 02139, United States

2 Center for Functional Nanomaterials, Brookhaven National Laboratory, Upton, NY 11973, USA

3 Department of Materials Science and Engineering, The University of Texas at Dallas, 800 West Campbell Road, Richardson, Texas 75080, USA

*jz2466@columbia.edu; englund@mit.edu

RIE Mask Creation by BCP Self-Assembly: To prepare the sample surface for BCP self assembly, we first clean the SiO_2 layer by oxygen plasma etching (March Plasma CS1701) for 1 minute at 20 W power with 100 mTorr pressure. To promote vertical orientation of self-assembled BCP domains, a “neutral” brush consisting of a hydroxyl-terminated polystyrene (PS) and poly(methyl methacrylate) (PMMA) random copolymer (PS-r-PMMA-OH, 61 mol. % styrene, determined by ^{13}C NMR, Mn = 9.2 kg/mol and Mw/Mn = 1.35, determined by gel permeation chromatography relative to PS standards), provided by The Dow Chemical Company (10.1063/1.5000965), is grafted to the SiO_2 surface by a dehydration reaction facilitated by baking the sample on a hot plate for 5 minutes at 250 °C in a nitrogen enriched environment. Ungrafted brush polymer is removed by subsequent spin-rinsing in propylene glycol monomethyl ether acetate (PGMEA). We then spin coat (3000 rpm) the BCP solution, 1% (w/w) in toluene, which is based on a cylindrical-phase polystyrene-b-poly(methyl methacrylate) (PS-b-PMMA) BCP (177 kg/mol, PS:PMMA = 131:46, Mw/Mn = 1.10, purchased from Polymer Source). The sample is thermally annealed on a hot plate for 20 minutes at 250°C in a N_2 enriched environment[1,2] to facilitate self-assembly. This self assembly process produces a hexagonal array of PMMA dots in a PS matrix as the thermodynamically favorable arrangement of the system, where the mean dot diameter is ~ 30 nm and the lattice period of the hexagonal array is ~ 70 nm.

Following self-assembly, SIS is used to selectively load the PMMA polymer domain with AlO_x via adsorption of trimethylaluminum (TMA) and H_2O sequentially for 4 cycles (100s dwell/purge times) in a commercial atomic layer deposition reactor (Cambridge Nanotech Savannah S100). After oxygen plasma ashing (March Plasma CS1701, 20 W power, 100 mTorr, 5 minutes) to

remove both the PS and PMMA polymers, a hexagonal array pattern of AlO_x dots remains and used as a hard masks for etching into SiO_2 layer and diamond. The form of the hexagonal array of AlO_x dots with ~ 30 nm in size and ~ 70 nm in pitch is shown in the SEM image in Figure 1(b). We then use SF_6 and oxygen plasma dry etching to transfer the hexagonal array dots pattern to the SiO_2 layer, as indicated in Figure 1(c). A directional oxygen plasma etching is performed to etch the diamond down to ~ 30 nm, which defines the height of the diamond nanocrystals. The resultant structures are diamond pillars with ~ 30 nm in height, which are shown in Figure 1(d) with side-view schematic and SEM images taken with the sample tilted by 45 degrees.

Isotropic Dry Etching for Undercutting Nanodiamond Pillars: With the size of the diamond nanocrystals defined by the size of the AlO_x dots and dwell time of the directional oxygen etching, it is necessary to undercut and release the diamond nanocrystals to complete the process. Undercutting nanostructures in fabrication has been realized by either homoepitaxial structure with sacrificial layers beneath the device layer, or engineered plasma etching with a Faraday cage[3,4] or zero RF driving power[5]. Here, we pursue the idea of quasi-isotropic etching for the release of diamond nanocrystals. Sidewall passivation with ~ 2 nm of conformal coating of ALD SiO_2 is performed to protect the diamond from the subsequent quasi-isotropic oxygen plasma etching, as shown in Figure 1(e). The ALD was performed in a viscous flow reactor using tris[dimethylamino]silane (3DMAS, 99.999%, Sigma-Aldrich) and ozone (O_3) as Si source and oxidant, respectively, at the process temperature of 250 °C. Growth thickness per cycle for the process was confirmed to be 1.3 Å/cycle. We used an exposure-mode ALD in which 1 ALD cycle consists of: 0.05 s dosing of 3DMAS, 28 s waiting under static vacuum, 13 s chamber purging (for removing excess precursors and reaction byproducts) under 100 sccm nitrogen flow, 0.2 s dosing of O_3, 7 s waiting under static vacuum, and, finally, 13 s chamber purging under 100 sccm nitrogen flow. Total 15 ALD cycles were applied to yield approximately 2 nm of SiO_2 conformal coating on etched diamond pillars. We then use a directional etching with a mixture of SF_6 and oxygen to selectively remove the SiO_2 on the non-sidewall surfaces, which opens up the bottom surfaces for the subsequent quasi-isotropic oxygen plasma etching of the diamond. We heat our samples at 90°C and slightly drive the plasma with ~ 5 W of biasing power, which gives a horizontal etch rate of ~ 0.5 nm/min at the sidewall surfaces with a vertical etch rate of ~ 2.5 nm/min. This undercut step enables the control over the size of the pedestal that the diamond nanocrystals sit on by timing the duration of the quasi-isotropic etching step. The diamond nanoparticles are undercut to nearly free-standing, so that they can be easily harvested. The final step is a HF acid immersion to remove the residue SiO_2 and AlO_x, after which we release the NDs on a ~130 μm thick glass coverslip for optical characterization. The release step is done by bringing the surface of the bulk IIA diamond in direct contact with the glass coverslip, and some NDs are attached to the surface of the the glass coverslip, possibly due to electrostatic binding.

Reference

1. Rahman, A. *et al.* Sub-50-nm self-assembled nanotextures for enhanced broadband

antireflection in silicon solar cells. *Nat. Commun.* **6**, 5963 (2015).

2. Doerk, G. S. & Yager, K. G. Rapid Ordering in 'Wet Brush' Block Copolymer/Homopolymer Ternary Blends. *ACS Nano* **11**, 12326–12336 (2017).

3. Burek, M. J. *et al.* Free-standing mechanical and photonic nanostructures in single-crystal diamond. *Nano Lett.* **12**, 6084–6089 (2012).

4. Schukraft, M. *et al.* Invited Article: Precision nanoimplantation of nitrogen vacancy centers into diamond photonic crystal cavities and waveguides. *APL Photonics* **1**, 020801 (2016).

5. Khanaliloo, B. *et al.* Single-Crystal Diamond Nanobeam Waveguide Optomechanics. *Phys. Rev. X* **5**, 041051 (2015).
